# Sociodemographic and clinical characteristics associated with overdose among patients with a substance-related diagnosis in the emergency department of Southern California

**DOI:** 10.1186/s12954-025-01233-9

**Published:** 2025-05-24

**Authors:** Brian Maila, María Luisa Zúñiga, Carla Marienfeld

**Affiliations:** 1 Department of Psychiatry, University of California, San Diego, 9500 Gilman Drive, C101, La Jolla, San Diego, CA 92093 USA; 2 School of Social Work, University of California, San Diego, 5500 Campanile Drive, San Diego, CA 92182 USA; 3 Chainama Hills College Hospital, Ministry of Health, P.0. Box 30043, Lusaka, Zambia

**Keywords:** Substance-related diagnosis, Substance use disorder, Emergency health services, Social determinants, Health disparities, Drug overdose, Mental health diagnosis

## Abstract

**Background:**

Drug overdoses are a significant public health concern, but there is limited research on the associated factors among patients presenting to the Emergency Department (ED). We investigated the sociodemographic and clinical characteristics associated with drug overdoses among patients with substance-related diagnoses (SRD) in a Southern California healthcare system’s ED.

**Methods:**

A cross-sectional study employed EPIC electronic medical records from a sample of 13,477 adults (18–90 years of age) diagnosed with an SRD ICD-10 classification and had an ED encounter within the UCSD Health system in Southern California during the period from January 1, 2020, to December 31, 2023. Bivariate and multiple logistic regression analyses were utilized to ascertain sociodemographic and clinical characteristics correlated with overdoses.

**Results:**

The odds of experiencing an overdose were higher among individuals with a cannabis-related diagnosis (aOR = 1.21, *p* < 0.05) in comparison to those lacking such a diagnosis, among individuals with an Opioid-related diagnosis (aOR = 1.14, *p* < 0.05) when compared to those without an Opioid-related diagnosis. Individuals aged 40–54 had higher odds (aOR = 1.37, *p* < 0.05) of experiencing an overdose compared to those aged 18–24. Additionally, the odds were more pronounced among Black or African American (aOR = 1.31, *p* < 0.001) individuals compared to whites, among individuals of Hispanic/Latinx origin (aOR = 1.22, *p* < 0.001) compared to those of non-Hispanic/Latinx origin, among those with public health insurance (aOR = 1.40, *p* < 0.001) compared to those with commercial health insurance, and among individuals with any mental health diagnosis (aOR = 1.13, *p* < 0.05) compared to those without such a diagnosis. In contrast, individuals experiencing overdose were less likely to be female (aOR = 0.78, *p* < 0.001) compared to male, and less likely to be married/living as married/having a significant other (aOR = 0.69, *p* < 0.001) compared to those who were single.

**Conclusion:**

Our research showed that individuals with cannabis or Opioid-related diagnoses had higher odds of experiencing an overdose. Patients with an overdose were typically middle-aged, Black or African American, and Hispanic/Latinx, had public health insurance, and had comorbid mental health diagnoses. They were less likely to be female, married, or in a significant relationship. These findings highlight the importance of sociodemographic and clinical factors in identifying at-risk individuals for targeted interventions.

**Supplementary Information:**

The online version contains supplementary material available at 10.1186/s12954-025-01233-9.

## Introduction

Drug overdose remains one of the leading causes of preventable death in the United States [1], with California experiencing a surge in recent years. Surveillance data indicate that overdose fatalities in the state have doubled over the past five years [2], driven largely by the proliferation of illicitly manufactured fentanyl (IMF) [3], and the increasing use of other substances, including stimulants, tranquilizers, and their combinations with synthetic opioids [2, 4–6]. In 2021 alone, California reported a 13.4% increase in emergency department (ED) visits for non-fatal drug overdoses across multiple drug classes compared to the previous year [7]. San Diego County mirrored this trend, recording 4,638 ED visits for drug-related overdoses—an increase of 15.7% from 4,128 visits in 2020 [7]. These escalating patterns of both fatal and non-fatal overdoses underscore the urgent need to better understand the characteristics of individuals at elevated risk. A more nuanced analysis of this population is essential to inform targeted prevention strategies, improve clinical care pathways, and guide evidence-based policy interventions.

The rising complexity of drug use patterns, particularly polysubstance use involving IMF, stimulants, anxiolytics, sedatives and hypnotics [8–13], has introduced new challenges to the overdose crisis by elevating the risk of fatal overdoses [14] and making it more challenging to treat overdoses. This trend highlights the complexity of the overdose epidemic, necessitating the need for a closer examination of the evolving patterns of overdoses and the impact of various drug classes beyond opioids [15] in the United States. Research has increasingly shown that overdose risk is not solely influenced by drug type or dosage, but also by a constellation of sociodemographic and clinical factors, including race and ethnicity, co-occurring mental health conditions, homelessness, recent incarceration, and limited access to healthcare services [16–18]. EDs serve as essential contact sites for those who use drugs, providing significant opportunities for intervention and the initiation of care. Nonetheless, predictive instruments capable of precisely stratifying overdose risk by utilizing real-world data are still inadequately developed and underused in clinical practice. Therefore, comprehending the characteristics of individuals presenting to the emergency department with overdoses is crucial for formulating appropriate triage, follow-up, and interventions.

Despite growing awareness of the overdose crisis, few studies have comprehensively analyzed the sociodemographic, clinical, and behavioral characteristics associated with drug overdose presentations in the ED, especially within specific local contexts like Southern California. Most existing surveillance systems track overdose trends in aggregate [19], offering limited insight into the profiles of individuals most affected. Moreover, epidemiological knowledge about these individuals remains limited mostly because prior research examining factors associated with non-fatal overdose has largely focused on examining opioid-related overdoses [20–28]. While the focus on opioid-related overdoses is warranted due to their significant impact, it restricts the applicability of study findings to individuals with overdoses from other substances such as cocaine, amphetamines, methamphetamines, cannabis, inhalants, sedatives, and poly-drug use. There is a critical need for localized, data-driven approaches to identifying high-risk populations and informing timely, targeted interventions. This study aims to fill that gap by examining the attributes of individuals who presented with a drug overdose diagnosis in the ED in Southern California during a time of rising overdose incidence.

In this study, we aim to describe and estimate the socio-demographic and clinical characteristics associated with drug overdose-related ED encounters among patients with SRDs from Southern California. Specifically, we seek to identify key factors associated with the likelihood of an overdose presentation. To this end, we hypothesize that individuals who are middle-aged, male, and those with co-occurring psychiatric conditions will have higher odds of presenting with a drug overdose. Additionally, we hypothesize that individuals diagnosed with opioid-related diagnosis will exhibit greater odds of overdose-related ED visits. These hypotheses are directly aligned with the overall aims of the study, which seek to inform risk stratification and guide clinical and public health responses to the ongoing overdose crisis.

## Materials and methods

### Sample and data source

This cross-sectional study utilized de-identified electronic health records of a total of 13,477 patients aged 18 to 90 with an ED encounter, who were in the substance-related diagnosis (SRD) registry of a large tertiary healthcare system in Southern California between January 1, 2020, and December 31, 2023. Inclusion in the registry is based on any SRD, which can include substance use disorders, substance intoxication, substance withdrawal, substance overdose, and substance use diagnoses. See supplemental Tables A and B for diagnosis categories and ICD codes included in the sample and used in the analyses. The SRD registry includes patients who have had encounters across the health system, including in primary care, specialty care, and the ED. The data collection process followed a formal request that went through a standardized data request process at the Altman Clinical and Translational Research Institute (ACTRI), including approval by the Institutional Review Board at the University of California, San Diego. ACTRI administered the request using The Data Extraction Concierge Service (DECS), which is managed by the health center’s biomedical informatics team. The Data Extraction and Concierge Service (DECS) retrieved the data from the EPIC Electronic Medical Records system at UCSD Health and provided it in a secured, Health Insurance Portability and Accountability Act (HIPAA)-approved Virtual Research Desktop.

### Measures

#### Dependent variable

Our dependent variable was a documented substance-related overdose, indicated as a binary dummy variable of any overdose diagnosis in the medical record for the patients during an ED visit in the 3-year period, January 1st, 2020, to December 31st, 2023, of data extraction (No/Yes). A new variable for substance-related overdose was recorded combining the ICD-codes in the dataset for each of the specific substance-related overdoses. The ICD 10-codes have been provided in the supplementary file.

#### Independent variables

The independent variables of our research were sociodemographic and clinical characteristics. The sociodemographic characteristics included age, gender, race, ethnicity, marital status, and health insurance. Age was treated as a categorical variable in five different groups, 18–24 years, 25–39 years, 40–54 years, 55–64 years, and 65–90 years. The combination sex variable was treated as a categorical variable for male, female, and other (including unknown, non-binary, choose not to disclose). Race was managed as a categorical variable grouped in seven groups namely Asian, American Indian, or Alaska Native, Black, or Africa American, Native Hawaiian or Pacific Islander, Other Mixed Race, White, and Uknown for those who chose not to disclose their race. Ethnicity was conceptualized as a categorical variable into three groups, Hispanic/Latinx, Not Hispanic/Latinx and unknown ethnic origin for those who chose not to disclose their ethnicity. Marital status was also assessed for and managed as a categorical variable in five different groups, namely single, married/living as married/significant other, divorced/separated, widowed, and unknown for those without a reported marital status. Health insurance was conceptualized as a categorical variable including five groups namely Medicare (Medicare/Medicare managed care), public (Medicaid-California/Medicaid-out-of-state/Medicaid managed care/County medical services), self-pay, other commercial (commercial/motor vehicle insurance/workers compensation/other managed care), and other government (Tricare/Federal Plans). The clinical characteristics examined in our analysis included medical comorbidity severity and mental health comorbidity. Medical comorbidity severity was managed as a binary variable, with those reporting no medical comorbidity having a Charlson Comorbidity Index = 0 and those reporting medical comorbidity having a Charlson Comorbidity Index ≥ 1 to reflect medical severity (ICD-10 codes available in the supplementary file) (Charlson et al., 1987). Mental health comorbidity was managed as a binary variable, named, any mental health diagnosis, and, coded with a value of 1 indicating “yes” and 0 indicating “no.” The variable was created by combining ICD-10 codes for specific mental health diagnosis (schizophrenia, schizotypal disorder, persistent delusional disorder, schizoaffective disorder, other psychotic disorder not caused by a substance or known physiological condition, unspecified psychosis, manic episode, bipolar disorder, or severe major depressive symptoms were considered as serious mental health diagnosis, to eating disorders, specific personality disorders, impulse disorders, obsessive-compulsive disorder, phobic anxiety disorder, other anxiety disorders, reaction to severe stress, mild or moderate major depressive disorder, and/or adjustment disorders), all managed as binary variables (yes (1)/ no (0)). The specific ICD-10 codes for each of the mental health diagnoses are included in the supplementary file. The specific substance-related diagnosis (SRDs) included alcohol (yes/no), other/poly substances (yes/no), opioids (yes/no), stimulants (yes/no), cannabis (yes/no), sedatives (yes/no), hallucinogen (yes/no), and inhalant (yes/no) that were all managed as binary variables.

#### Statistical analysis

Descriptive statistical analysis was used to characterize the sample, with the results reported as means for continuous variables, proportions, and frequencies for categorical and binary variables. Univariate analysis was conducted using Chi-squared (X²) tests of significance for categorical data to examine group differences, reporting the proportions, percentages, and the corresponding p-values at the 5% level of significance. Bivariate logistic regression was then conducted to estimate the associations between each of the independent variables and a substance-related overdose diagnosis, reporting the unadjusted odd ratios (ORs) with the corresponding p-values at 5% significance level. Thereafter, a multivariable logistic regression was conducted between all the independent variables [age, gender, race, ethnicity, marital status, health insurance, Charlson comorbidity, any mental health diagnosis, specific SRDs (alcohol, other/poly substances, opioids, stimulants, cannabis, sedatives, hallucinogen, and inhalant)] and substance-related overdose diagnosis, reporting the adjusted odd ratios (ORs) with the corresponding p-values at 5% significance level. Any variables with OR > 1 and *P* < 0.05 were considered to have a statistically significant association with an overdose diagnosis and were more likely to present with an overdose diagnosis to the ED. Any ORs < 1 and *P* < 0.05 were considered to have a statistically significant association with a reduced likelihood of presenting with an overdose diagnosis to the ED.

## Results

### Description of the sample included in the analysis

**Fig. 1:**
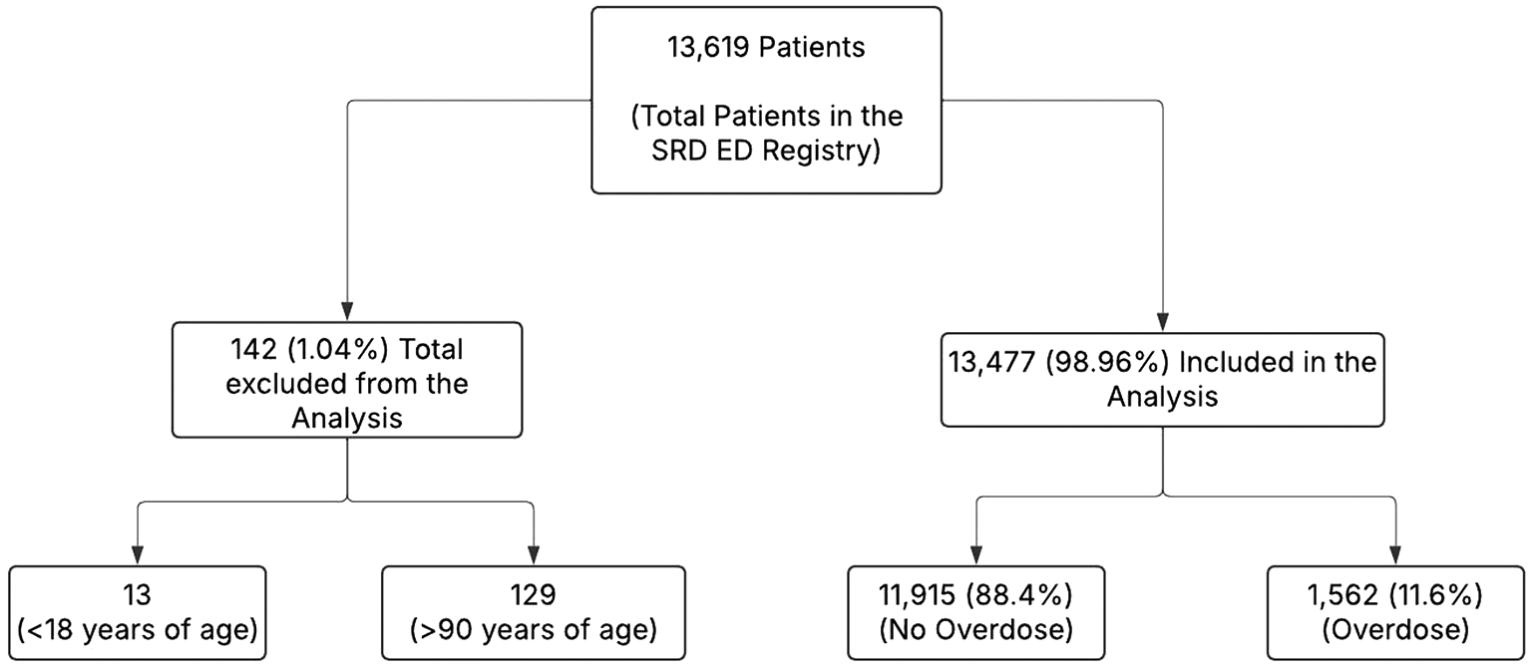
Flowchart for the sample included in the study

Data for a total of 13,619 patients with an ICD-10 SRD diagnosis were identified during emergency room (ED) encounters from January 1st, 2020, to December 31st, 2023. Of these, 0.01% (129) were excluded due to age being above 90 years, and 0.001% were excluded due to age being below 18 years (Refer to Fig. 1).

### Sociodemographic and clinical characteristics of the sample

A significant portion of these patients were male (65.1%), white (59.1%), Hispanic/Latinx (73.9%), single (63.4%), and possessed commercial/motor vehicle insurance/workers compensation/other managed care for health insurance (48%). The average age of the overall study sample was 49.42 (SD = 16.06). Among the total sample, 5071 individuals (37.2%) had any mental illness, while 4944 individuals (36.7%) had a Charlson Comorbidity Index of 1 or higher (Refer to Table 1). Among 13,477 patients diagnosed with a SRD, 1,562 (11.6%) experienced an overdose. A significant portion of individuals who experienced overdoses were male (12.7%), identified as not Hispanic/Latinx (12.0%), Black or African American (14.9%), single (12.7%), and utilized Medicaid-California/Medicaid-Out-of-State/Medicaid Managed Care/County Medical Services health insurance (15.4%). The mean age of the patients with an overdose in this study was 48.30 (SD = 15.31) years. Individuals with any mental health diagnosis experienced a higher frequency of overdoses (12.6%). The analysis revealed that individuals with a Charlson comorbidity score of ≥ 1 and those with a score of = 0 had comparable frequencies of overdoses (Refer to Table 1).

**Table 1:** Characteristics of the patients in the emergency department with an SRD of a health system in Southern California, stratified by the presence or absence of a substance-related overdose

Characteristic	Total Sample	Overdose Diagnosis	No Overdose Diagnosis	*P*-Value
**All** (n [%])	13,477 (100)	1562 (11.6)	11,915 (88.4)	
**Age (range 18–90; M ± SD)**	49.42 ± 16.06	48.30 ± 15.31	49.57 ± 16.15	
**Age Categories** (n [%])				
18–24 years	564 (4.2)	54 (9.6)	509 (90.4)	*P* < 0.001
25–39 years	3894 (28.9)	477 (12.2)	3417 (87.8)	
40–54 years	3501 (26.0)	459 (13.1)	3042 (86.9)	
55–64 years	2733 (20.3)	301 (11.0)	2432 (89.0)	
65–90 years	2787 (20.7)	271 (9.7)	2515 (90.3)	
**Sex (n [%])**				*P* < 0.001
Male	8771 (65.1)	1114 (12.7)	7657 (87.3)	
Female	4695 (34.8)	446 (9.5)	4249 (90.5)	
Unknown	11 (0.1)	2 (18.2)	9 (81.8)	
**Race (n [%])**				
White	7969 (59.1)	889 (11.2)	7080 (88.8)	*P* < 0.001
Asian	302 (2.2)	25 (8.3)	277 (91.7)	
American Indian or Alaskan Native	109 (0.8)	12 (11.0)	97 (89.0)	
Black or African American	1622 (12)	242 (14.9)	1380 (85.1)	
Native Hawaiian or Pacific Islander	45 (0.3)	3 (6.7)	42 (93.3)	
Other Mixed Race	3276 (24.3)	388 (11.8)	2888 (88.2)	
Unknown*	150 (1.1)	3 (2.0)	147 (98.0)	
**Ethnicity Hispanic/Latinx (n [%])**				
Hispanic/Latinx	9958 (73.9)	327 (10.8)	2698 (89.2)	*P* < 0.001
Not Hispanic/Latinx	3025 (23.0)	1191 (12.0)	8767 (88.0)	
Unknown Ethnic Origin**	165 (1.3)	6 (3.6)	159 (96.4)	
**Marital Status (n [%])**				
Single	8544 (63.4)	1086 (12.7)	7458 (87.3)	*P* < 0.001
Married, living as married, significant other	2738 (20.3)	227 (8.3)	2511 (91.7)	
Divorced/separated	1750 (13.0)	211 (12.1)	1539 (87.9)	
Widowed	420 (3.1)	37 (8.8)	383 (91.2)	
Unknown	15 (0.1)	1 (6.7)	14 (93.3)	
**Health Insurance** (**n [%])**				
Other Commercial (Commercial/motor vehicle insurance/workers compensation/other managed care)	6466 (48)	67 (10.5)	5787 (89.5)	
Medicare (Medicare/Medicare managed care)	1757 (13)	188 (10.7)	1569 (89.3)	*P* < 0.001
Public (Medicaid-California/Medicaid-out-of-state/Medicaid managed care/county medical services)	2429 (18)	373 (15.4)	2056 (84.6)	
Self-Pay	2571 (19.1)	295 (11.5)	2276 (88.5)	
Other government (Tricare/Federal Plans)	254 (1.9)	27 (10.6)	227 (89.4)	
**Any Mental Illness**				
Yes	5071 (37.2)	641 (12.6)	4430 (87.4)	*P* < 0.05
No	8548 (62.8)	921 (11.0)	7485 (89.0)	
**Charlson Comorbidity (n [%])**				
Charlson Comorbidity Index ≥ 1	4944 (36.7)	573 (11.6)	4371 (88.4)	*P* > 0.05
0 Charlson Comorbidity Index = 0	8533 (63.3)	989 (11.6)	7544 (88.4)	

### Associations between sociodemographic factors, clinical characteristics and any substance-related overdose diagnosis

Based on the adjusted model individuals aged 40–54 had higher odds (aOR = 1.37, CI = 1.01–1.87, *p* < 0.05) of experiencing an overdose when compared to those aged 18–24. Female patients exhibited reduced odds (aOR = 0.76, CI = 0.69–0.87, *p* < 0.001) of encountering an overdose in comparison to their male counterparts. Individuals identifying as Black or African American exhibited higher odds (aOR = 1.31, CI = 1.12–1.53, *p* < 0.001) of experiencing an overdose in comparison to their white counterparts. Individuals who are Hispanic/Latinx had higher odds (aOR = 1.22, CI = 1.03–1.44, *p* < 0.05) of experiencing an overdose when compared to their non-Hispanic/Latinx counterparts. Individuals in married, living together as married, or with significant other had notably reduced odds (aOR = 0.69, CI = 0.59–0.81, *p* < 0.001) of experiencing an overdose when compared to single individuals. People with public health insurance, like Medicaid or County medical services, had higher odds (aOR = 1.40, CI = 1.22–1.61, *p* < 0.001) of experiencing an overdose compared to those that had commercial health insurance. People diagnosed with a cannabis substance-related diagnosis had higher odds (aOR = 1.21, CI = 1.02–1.44, *p* < 0.05) of experiencing an overdose than those who did not have this diagnosis. Individuals with opioid substance-related diagnoses had higher odds (aOR = 1.14, CI = 1.003-1.30, *p* < 0.05) of experiencing an overdose in comparison to those without such a diagnosis (Refer to Table 2).

**Table 2:** Associations between sociodemographic factors, clinical characteristic, specific substance-related diagnosis and substance-related overdose diagnosis

	Bivariate (Unadjusted Models)					Multivariable (Adjusted Model)				
Characteristic	B	Se	OR	95% CL	*P*-Value	B	Se	aOR	95% CI	*P*-Value
**Age Categories**										
18–24 years	Ref	Ref	-	-	-	Ref	-	-	-	-
25–39 years^**^	0.274	0.151	1.32	0.98–1.77	> 0.05	0.221	0.156	1.25	0.92–1.69	> 0.05
40–54 years	0.352	0.152	1.42	1.06–1.91	< 0.05	0.315	0.157	1.37	1.01–1.87	< 0.05
55–64 years	0.154	0.156	1.17	0.86–1.58	> 0.05	0.123	0.163	1.13	0.82–1.56	> 0.05
65–90 years	0.016	0.157	1.02	0.75–1.38	> 0.05	0.044	0.170	1.05	0.75–1.46	> 0.05
**Sex**										
Male	Ref	-	-	-	-	Ref	-	-	-	-
Female^**^	-0.326	0.059	0.72	0.64–0.81	< 0.001	-0.255	0.061	0.76	0.69–0.87	< 0.001
Unknown	0.424	0.782	1.53	0.33–7.08	> 0.05	0.330	0.786	1.39	0.30–6.49	> 0.05
**Race**										
White	Ref	-	-	-	-	Ref	-	-	-	-
Asian	-0.330	0.212	0.72	0.48–1.09	> 0.05	-0.327	0.217	0.72	0.47–1.11	> 0.05
American Indian or Alaskan Native	-0.015	0.308	0.99	0.54–1.8	> 0.05	0.031	0.310	1.03	0.56–1.89	> 0.05
Black or African American^**^	0.334	0.078	1.40	1.20–1.63	< 0.001	0.267	0.080	1.31	1.12–1.53	< 0.001
Native Hawaiian or Pacific Islander	-0.564	0.599	0.57	0.18–1.84	> 0.05	-0.546	0.601	0.58	0.18–1.88	> 0.05
Other Mixed Race	0.068	0.065	1.07	0.94–1.22	> 0.05	0.118	0.084	1.13	0.95–1.33	> 0.05
Uknown^**^	-1.827	0.584	0.16	0.05–0.51	< 0.05	-1.459	0.601	0.23	0.07–0.76	< 0.05
**Ethnicity Hispanic/Latinx**										
Not Hispanic/Latinx	Ref	-	-	-	-	Ref	-	-	-	-
Hispanic/Latinx^**^	0.114	0.066	1.12	0.98–1.28	> 0.05	0.195	0.087	1.22	1.03–1.44	< 0.05
Unknown Ethnic Origin	-1.167	0.420	0.31	0.14–0.71	< 0.05	-0.603	0.436	0.55	0.23–1.29	> 0.05
**Marital Status**										
Single	Ref	-	-	-	-	Ref	-	-	-	-
Married, living as married, significant other^**^	-0.477	0.077	0.62	0.53–0.72	< 0.001	-0.370	0.082	0.69	0.59–0.81	< 0.001
Divorced/separated	-0.060	0.080	0.94	0.80–1.10	> 0.05	0.037	0.086	1.04	0.88–1.23	> 0.05
Widowed	-0.410	0.175	0.66	0.47–0.94	< 0.05	-0.189	0.183	0.83	0.58–1.18	> 0.05
Unknown	-0.712	1.036	0.49	0.06–3.73	> 0.05	0.083	1.065	1.09	0.14–8.76	> 0.05
**Health Insurance**										
Other Commercial (Commercial/Motor vehicle insurance/Workers compensation/other managed care)	Ref	-	-	-	-	Ref	-	-	-	-
Medicare (Medicare/Medicare managed care)	0.021	0.087	1.02	0.86–1.21	> 0.05	0.143	0.097	1.15	0.95–1.40	> 0.05
Public (Medicaid-California/Medicaid-out-of-state/Medicaid managed care/County medical services)^**^	0.436	0.069	1.55	1.35–1.77	< 0.001	0.335	0.071	1.40	1.22–1.61	< 0.001
Self-Pay	0.100	0.074	1.11	0.96–1.28	> 0.05	0.086	0.076	1.09	0.94–1.27	> 0.05
Other Government (Tricare/Federal Plans)	0.014	0.208	1.01	0.68–1.52	> 0.05	0.111	0.210	1.12	0.74–1.69	> 0.05
**Any Mental Illness**										
No	Ref	-	-	-	-	Ref	-	-	-	-
Yes^**^	0.162	0.055	1.18	1.06–1.31	< 0.05	0.122	0.058	1.13	1.01–1.26	< 0.05
**Charlson Comorbidity**										
Charlson Comorbidity Index = 0	Ref	-	-	-	-	Ref	-	-	-	-
Charlson Comorbidity Index ≥ 1	0.000	0.056	1.00	0.90–1.12	> 0.05	-0.022	0.058	0.98	0.87–1.10	> 0.05
**Specific Substance-Related Diagnosis**										
Alcohol related diagnosis										
No	Ref	-	-	-	-	Ref	-	-	-	-
Yes	-0.001	0.057	1.00	0.89–1.12	> 0.05	0.055	0.065	1.06	0.93–1.20	> 0.05
Opioid related diagnosis										
No	Ref	-	-	-	-	Ref	-	-	-	-
Yes^**^	0.076	0.058	1.08	0.96–1.21	> 0.05	0.132	0.066	1.14	1.003-1.30	< 0.05
Sedative Hypnotic Anxiolytic Related Diagnosis										
No	Ref	-	-	-	-	Ref	-	-	-	-
Yes	0.209	0.150	1.23	0.92–1.66	> 0.05	0.174	0.156	1.19	0.88–1.61	> 0.05
Cannabis related diagnosis										
No	Ref	-	-	-	-	Ref	-	-	-	-
Yes^**^	0.212	0.081	1.24	1.05–1.45	< 0.05	0.191	0.088	1.21	1.02–1.44	< 0.05
Stimulant related diagnosis										
No	Ref	-	-	-	-	Ref	-	-	-	-
Yes	0.007	0.058	1.01	0.90–1.13	> 0.05	0.011	0.059	1.01	0.90–1.14	> 0.05
Inhalant related diagnosis										
No	Ref	-	-	-	-	Ref	-	-	-	-
Yes	0.646	0.791	1.91	0.41–8.99	> 0.05	0.598	0.798	1.82	0.38–8.68	> 0.05
Other Psychoactive substance related diagnosis										
No	Ref	-	-	-	-	Ref	-	-	-	-
Yes	0.006	0.067	1.01	0.88–1.15	> 0.05	0.042	0.071	1.04	0.91–1.20	> 0.05

**Indicates significant associations with a substance-related overdose diagnosis

## Discussion

This research aimed to examine the socio-demographic and clinical characteristics associated with drug overdoses among people presenting to the emergency department with a substance-related diagnosis in Southern California. The analysis indicated that individuals with a substance-related disorder (SRD) aged 40 to 54, identifying as Black or African American, of Hispanic/Latinx origin, utilizing public health insurance, reporting any mental health diagnosis, and having an opioid or cannabis-related diagnosis exhibited increased odds of presenting with an overdose to the emergency department (ED). The study indicated that females, as well as individuals who are married, living as married, or in a significant relationship, exhibited lower odds of presenting with an overdose during their emergency department visit. The findings align with the hypothesis of this study. The following is a detailed analysis of these significant findings.

In our Southern California study, individuals aged 40 to 54 had higher odds of presenting with an overdose in the emergency department, aligning with previous U.S. research showing increased overdose rates in those aged 45 to 54 [29] and 45 to 59 [30]. Globally, age-related overdose trends vary. A study in Osaka reported that individuals aged 16 to 40 had higher odds of being transported to the ER for overdoses [31], while a Tokyo study reported the highest overdose odds in those aged 65 and older, followed by those aged 15 to 34 and 35 to 64 [32]. In Jeddah, children under 12 had the highest odds of overdose [33]. Despite these global differences, our findings emphasize the increasing concern of overdoses among middle-aged individuals, highlighting the need for harm reduction interventions specifically tailored to this age group in emergency departments.

Our study found higher odds of overdose among individuals identifying as Black or African American and Hispanic/Latinx, aligning with recent trends showing a higher overdose burden in these groups compared to White non-Hispanic counterparts [16]. One study reported that nearly 2% of Black men aged 45 experience an overdose before 60, compared to 1% of White men [34]. Overdose fatalities among Black men aged 45 to 59 also exceeded those of their White counterparts [34]. Recent analyses highlight a disproportionate rise in Opioid-related overdoses among Black individuals compared to whites [35], linked to factors such as socio-economic changes from the COVID-19 pandemic, differing polysubstance use patterns, increased fentanyl exposure, and inequities in treatment access and naloxone distribution [36]. These findings underscore the need for overdose interventions addressing the social determinants and structural factors contributing to racial disparities perpetuating drug use and consequent overdoses.

Our study found that individuals with public health insurance had higher odds of presenting with an overdose at the ED, consistent with previous research showing higher overdose death rates among Medicaid beneficiaries, which were twice as high as those for all U.S. residents [37]. The ED sample, consisting of approximately 74% Hispanic/Latinx individuals, reflects disparities in health insurance coverage, as Latinos are more likely to be uninsured or rely on public health insurance, often seeking care in the ED as a last resort [38]. Despite progress in reducing coverage disparities, Latino/x Californians remain uninsured at rates three times higher than White Californians, with Black and Asian Californians also experiencing higher uninsured rates double those of White Californians [38]. These findings underscore the need for continued monitoring of overdose trends to ensure that prevention and treatment efforts for SRDs are not disrupted by public health crises like the COVID-19 pandemic [39].

The present research indicates that individuals identifying as female have lower odds of presenting with an overdose to the ED. However, previous studies offer a more complex picture, with some suggesting higher overdose rates among females, particularly in relation to prescription medication overdoses [40], while others indicate lower rates in illicit Opioid-related overdoses [41]. These discrepancies may be due to varying patterns of substance use, healthcare access, and demographic factors across genders [42], as well as differences in the substances involved and geographic locations.

Our research supports prior findings that relationship status influences overdose risk [43], with married individuals showing lower odds of overdose compared to single individuals. Marriage may offer a protective effect through stronger social support, emotional assistance during stress, and stability, all of which promote healthier choices due to marital responsibilities, reduce substance use and prevent overdoses [44]. The financial and emotional benefits of marriage may also reduce economic stress and substance use as a coping mechanism [45]. These factors collectively contribute to the lower incidence of drug overdoses among married individuals, though individual experiences may vary. Thus, programs targeting overdose prevention should consider bolstering social support for individuals with an SRD.

Our research shows that individuals with a mental health diagnosis have significantly higher odds of presenting to the ED with drug overdoses, consistent with prior studies. A Canadian study found that recent overdose events increased the likelihood of mental health diagnoses and emergencies among drug overdose hotline users [46]. Similarly, a U.S. study linked unmet mental health needs and psychological pain to increased odds of non-fatal overdoses among individuals with Opioid-related diagnoses [47]. Another study reported that past-year mental distress was associated with higher odds of experiencing and witnessing overdoses among people who inject drugs [48]. These findings reflect greater exposure to drug use opportunities and disparities in accessing mental health and substance use treatment services for those with co-occurring disorders [49, 50]. Our results highlight the need to remove barriers and support policies and funding that integrate mental health and SRD interventions to reduce overdose rates.

The present study found that individuals with Opioid-related diagnoses had higher odds of presenting with overdoses in the ED, consistent with prior research. For example, a U.S. study reported higher ED encounter rates for Opioid-related diagnoses, with death rates of 69.2 per 10,000 patients—six times higher than for other ED patients [51]. Additionally, our findings align with another U.S. study identifying heroin and non-heroin opioid overdoses, including polysubstance use, as major contributors to overdose-related ED visits [52]. We also found that individuals with cannabis-related diagnosis had higher odds of presenting to the ED with an overdose, a relationship not yet definitively established in U.S. research. While previous studies suggest that cannabis use among older adults increases ED visits due to injury, it does not correlate with higher overdose rates compared to other substances [53]. Other researchers have reported that marijuana legalization in Massachusetts led to increased cannabis-related ED visits, marked by higher THC levels and cannabis-related ICD-10 codes, though it did not compare cannabis-related ED visits to those for other substances [54]. Our findings support trends linking cannabis use to ED visits due to adverse effects [55]. Future research should explore this association with larger sample sizes and longitudinal designs to establish the directionality of the relationship between cannabis-related ED visits and overdoses, informing targeted prevention for individuals at risk of developing cannabis related diagnosis by identifying modifiable risk factors.

### Limitations and strengths of the study

Our study has several limitations. First, the cross-sectional design prevents causal inferences, as we could not establish directionality between the independent and dependent variables. Additionally, we lacked longitudinal data and could not conduct overtime analysis to examine the sequence from SRD to overdose occurrence, hindering our ability to establish temporality in our sample. However, our study provides important insights and adds to the body of knowledge on overdoses. Specifically, our study grouped various substance-related overdoses into a single overdose category, contributing to limited work in this area, as prior studies have primarily focused on Opioid-related overdoses. Our study further provides information on where we need to go for subsequent research to inform treatment and overdose prevention interventions. Future research should incorporate longitudinal data to establish temporal relationships between SRDs and overdoses. Furthermore, our sample was drawn from an ED population, which may not represent individuals who experience overdoses at community level but do not seek ED services. This limitation may affect the generalizability of our findings to the broader population. However, examining factors associated with overdose-related ED encounters may inform interventions for post-discharge planning for patients at risk of repeat overdoses. Another limitation was missing data on gender identity, leading to the exclusion of this variable. As a result, the present study did not capture insights of overdoses based on gender identities. Future research should endeavor to capture this variable to give important insights that can inform targeted overdose prevention interventions for individuals with various gender identities. Additionally, the clinical database used in our study may be prone to misclassification or underreporting of SRDs and overdoses, as some diagnoses may be recorded under other psychoactive substances or omitted entirely. Furthermore, the substance specific diagnosis categories were not mutually exclusive. The participants belonging to categories like alcohol, stimulants, cannabis, inhalant and other psychoactive substances may have belonged to more than one substance-specific category, a feature not accounted for in the available dataset. This fails to give a comprehensive picture of polysubstance use patterns and introduces ambiguity and complexity in the interpretation of data. The overlap in the categories may introduce challenges in distinguishing the associations reported for each substance in our research. Therefore, future research should utilize mutually exclusive categories to improve clarity and enhance the precision of the associations between substance-specific diagnoses and overdose. The dataset lacked a distinction between medical and non-medical cannabis usage. As such, we were unable to determine whether cannabis-related diagnoses associated with overdose reflected prescribed medical cannabis, recreational use, or other sources. Future investigations ought to focus on exploring these differences to enhance comprehension of the context surrounding cannabis-related overdose risk. In addition, the dataset utilized in our research did not encompass information concerning the diverse patterns of polysubstance use among patients seeking treatment at the emergency department. Therefore, our study did not examine the associations between polysubstance use and overdoses. Future investigations should examine the associations between polysubstance use and presenting to the ED with an overdose in Southern California. Our study found no association between Charlson medical comorbidity and overdoses, possibly confounded by unassessed prior primary care visits. The lack of data on prior healthcare utilization means we could not fully capture patients’ health status before ED services, potentially impacting the Charlson comorbidity index. Future research should include prior healthcare utilization to better understand service types, quality, and health outcomes prior to ED utilization for management overdoses among the patients included in our study. The present study did not include sensitivity analyses and assessment of potential effect modification by important demographic or clinical factors, which may lead to the omission of significant variations in the relationship between risk factors and overdoses among different subpopulations. Subsequent research ought to investigate these elements to enhance comprehension of varying risk and fortify the reliability of results. Consequently, readers should exercise caution when drawing general conclusions based on the findings of our study.

### Study implications

This study has important implications for clinical practice, public health policy, tertiary prevention, and healthcare administration. The findings highlight that EDs are not only sites for acute care but also critical hubs for public health efforts to reduce morbidity and mortality related to SRDs. By using demographic and clinical data, healthcare providers can better identify at-risk individuals, design interventions to prevent repeat overdoses, and facilitate access to addiction treatment and harm reduction services for patients with SRDs managed in the ED [56–59]. Thus, policies prioritizing comprehensive SRD screening, the provision of naloxone, and the development of referral pathways for treatment could further mitigate the high rates of ED revisits due to overdoses [39, 60, 61]. Furthermore, the findings advocate for a shift in overdose prevention strategies. Rather than focusing solely on reducing access to or demand for psychoactive substances, interventions should address the root causes of overdose through comprehensive programs, policy initiatives, and harm reduction approaches. These may include ED-based screening and referral services [62], warm handoff programs to community-based care [63, 64], distribution of naloxone and fentanyl test strips [65, 66], initiation of medications for substance-related disorders (SRDs) in the ED [67, 68], and robust post-discharge follow-up [69, 70]. Additionally, data-driven early warning systems that flag individuals at high risk of overdose—based on prior ED encounters—can support proactive outreach and coordinated care by multidisciplinary teams [71–73]. Our results support developing interventions targeting the socio-economic and structural determinants of overdoses. EDs can play a crucial role in linking overdose patients to services that enhance economic stability, improve job prospects, foster social cohesion, reduce isolation, build resilience, combat racial stereotyping, promote equity in healthcare, improve access to mental health support, and engage communities in designing services to improve overall well-being [71]. Additionally, these findings can inform capacity-building programs for healthcare providers, equipping them with knowledge and skills on the social determinants of overdoses to better address patient needs [71]. Our study identifies sociodemographic and clinical factors associated with overdoses, laying the groundwork for future research on population-level and substance-specific factors to inform interventions [74].Future research should explore barriers, facilitators, and strategies for programs that enhance access to education, employment, and housing stability for patients with SRDs to address the overdose crisis in the U.S. [74]. Additionally, research should focus on reducing stigma and discrimination to improve care access for individuals with SRDs [74]. Further studies should examine structural factors influencing treatment-seeking behaviors, such as legal frameworks and the impact of criminalization versus medicalization on treatment availability [74]. Needs assessments for treatment and harm reduction initiatives in Black or African American communities are crucial to identify care gaps and inform the establishment of services close to those in need [74]. Researchers should also consider task-sharing approaches, leveraging community networks like peer navigators and social prescribing to promote health and prevent relapse among patients discharged from the ED after overdose treatment [74]. Addressing the structural barriers to treatment through research can improve health outcomes and quality of life, ultimately mitigating the overdose crisis.

## Conclusion

Our research provides valuable insights into the sociodemographic and clinical factors associated with overdoses among patients with SRDs presenting to the ED. Demographically, individuals aged 40 to 54, Black or African American, Hispanic/Latinx, and those with public health insurance had higher odds of experiencing and presenting with an overdose. In contrast, females and individuals who were married, living as married, or with a significant other had lower odds of presenting with an overdose during the study period. Clinically, individuals with comorbid mental health diagnoses, Opioid-related diagnoses, and cannabis-related diagnoses were more likely to present to the ED with an overdose. These findings highlight the complex interplay of sociodemographic and clinical factors contributing to overdoses among patients with SRDs, emphasizing the need for targeted interventions to reduce overdose risk and improve treatment outcomes for these patients.

### Future research directions

Future research should include prior healthcare utilization to better understand the types and quality of services received, as well as health outcomes prior to ED overdose encounters. Investigations examining the associations between polysubstance use and overdose-related ED visits are also warranted, given the growing complexity of substance use patterns. To improve clarity and enhance the precision of substance-specific associations, future studies should consider using mutually exclusive diagnostic categories. Additionally, efforts should be made to capture data on gender identity to provide insights that can inform targeted overdose prevention interventions for individuals with diverse gender identities. Incorporating longitudinal data will be critical to establishing temporal relationships between SRDs and overdose events. Future research should explore potential effect modification by key demographic and clinical factors to better identify subgroups at elevated risk of drug overdose. Conducting sensitivity analyses across these dimensions may provide deeper insight into the heterogeneity of risk and improve the precision of targeted interventions. Additionally, future studies should distinguish between medical and non-medical cannabis use, as these patterns may carry differing implications for overdose risk. Clarifying these distinctions is essential for contextualizing the role of cannabis use in polysubstance overdose and for informing nuanced clinical guidance and evidence-based policy decisions.

## Electronic supplementary material

Below is the link to the electronic supplementary material.

Supplementary Material 1

## Data Availability

No datasets were generated or analysed during the current study.

## Abbreviations

IMF: Illicitly Manufactured Fentanyl
SRD: Substance-related diagnosis
SRDs: Substance-related diagnoses
SUD: Substance Use Disorder
SUDs: Substance Use Disorders
ED: Emergency department
HIPAA: Health Insurance Portability and Accountability Act
IRB: Kuali Institutional Review Board (IRB)
EMR: Electronic Medical Records
